# Clinical evaluation of an automated TSI bridge immunoassay in the diagnosis of Graves’ disease and its relationship to the degree of hyperthyroidism

**DOI:** 10.1186/s12902-022-01114-3

**Published:** 2022-08-31

**Authors:** Tianqi Liu, Xiuying Zhang, Li Long, Lingli Zhou, Jing Chen, Meng Li, Ying Gao, Xianghai Zhou, Xueyao Han, Linong Ji

**Affiliations:** 1 Department of Endocrinology and Metabolism, Peking University People’s Hospital, N0.11, Xi Zhi Men Nan Da Jie, Xicheng District, Beijing, 100044 China; 2 Department of Endocrinology and Metabolism, the Second People’s Hospital, Guiyang, 550000 China

**Keywords:** TSH receptor antibody, Graves’ disease, Hyperthyroidism

## Abstract

**Background:**

The rapid and accurate detection of thyroid-stimulating hormone (TSH) receptor antibodies has always been an urgent need for the clinical diagnosis and management of Graves’ disease (GD). We aimed to evaluate the use of an automated thyroid-stimulating immunoglobulin (TSI) bridge immunoassay in the diagnosis of GD and to analyze the relationship between TSI and the degree of hyperthyroidism.

**Methods:**

A total of 227 new-onset GD patients, 29 Hashimoto thyroiditis, 43 non-autoimmune thyroid diseases and 37 euthyroid controls were consecutively recruited. All participants accepted the measurement of their serum thyroid function and thyroid-associated antibodies, including TSI being measured by an Immulite 2000 bridge immunoassay and TSH receptor autoantibodies (TRAb) being measured by a third-generation Roche electrochemiluminescence immunoassay. The quantitative consistency between the TSI and TRAb detection methods was analyzed by using Passing-Bablok regression and Bland–Altman plots. The diagnostic performance for GD was assessed by receiver operating characteristic (ROC) curve analysis.

**Results:**

Among 227 GD patients (174 females and 53 males, with a mean age of 39 years), the quantitative TSI was positively correlated with TRAb (*r* = 0.8099). According to the cut-off values proposed by the manufacturers (TSI: 0.55 IU/L, TRAb: 1.75 IU/L), the positive rates of TSI and TRAb in new-onset GD patients were 96.92% and 95.15%, respectively. Both TSI and TRAb levels positively correlated with FT_4_ levels (TSI: *r* = 0.243, TRAb: *r* = 0.317; all *P* < 0.001) and FT_3_ levels (TSI: *r* = 0.288, TRAb: *r* = 0.360; all *P* < 0.001) in new-onset GD patients. The ROC analysis showed that the optimal TSI cut-off value was 0.577 IU/L for GD diagnosis in this Chinese population, with a sensitivity of 96.92% and a specificity of 97.25%, respectively. The optimal TRAb cut-off value of was 1.38 IU/L, with a sensitivity of 96.92% and a specificity of 99.08%. There were no significant differences between the cut-off values obtained through the ROC analysis and those provided by the manufacturer for both TSI and TRAb when calculating their sensitivity and specificity in diagnosing GD. Among the 8 newly diagnosed GD cases with discordant qualitative antibody results, TSI was more likely than TRAb to match the clinical diagnosis of GD (6 TSI-positive vs. 2 TRAb-positive patients).

**Conclusion:**

The automated TSI bridge immunoassay was positively correlated with thyroxine levels in new-onset GD patients and was more likely to be consistent with the clinical diagnosis of GD than with that of TRAb. The positive Immulite 2000 TSI cut-off value of 0.577 IU/L for GD diagnosis in the Chinese population were close to the value recommended by the manufacturer.

**Supplementary Information:**

The online version contains supplementary material available at 10.1186/s12902-022-01114-3.

## Introduction

Graves’ disease (GD) is the most common cause of hyperthyroidism. It usually occurs in women of childbearing age and is one of the main factors that contribute to adverse pregnancy outcomes [1, 2]. The pathogenesis of GD is not yet clear. It is generally accepted that GD is an autoimmune disease caused by the combined action of genetic and environmental factors [3, 4]. A representative autoimmune feature of GD is the presence of autoantibodies known as thyroid-stimulating hormone (TSH) receptor antibodies, which can stimulate thyroid follicular cells to produce excess thyroid hormone, thus inducing a variety of hypermetabolic symptoms and characteristic signs, such as exophthalmos and anterior tibial mucinous oedema [5]. GD not only significantly reduces quality of life and work ability but also increases the risk of multiple complications and death [6]. Therefore, timely and accurate diagnosis is essential for the clinical management of GD patients.

As a pathogenic antibody, the TSH receptor autoantibody (TRAb) is very important in the diagnosis and treatment of GD [7, 8]. However, TRAb has not been formally incorporated into the essential diagnostic criteria of GD hyperthyroidism. One of the reasons for this is that the TRAb detection method has not been globally unified. Second, although a third-generation automated TRAb detection method with high sensitivity and specificity has been successfully developed and used in clinical practice [9], it still cannot effectively identify the stimulating antibody type, thyroid stimulating immunoglobulin (TSI), which is the GD-specific pathogenic antibody. Therefore, researchers have been committed to exploring TSI detection methods. In the past three decades, there have been at least three generations of updates for TSI detection methods [10, 11]. The representative method of the first-generation assays used human thyroid cell monolayers incubated with patients' sera and measured the production of cAMP [12]. Then, Chinese hamster ovary (CHO) cells transfected with the human TSH receptor were developed to increase the TSI sensitivity [13]. Recently, a new luciferase reporter-based chimeric receptor assay was introduced as the third-generation assay [14]. Although the cAMP-responsive TSI bioassay has a high accuracy for GD diagnosis, the detection process is complicated and time-consuming, and it is difficult to perform automation or batch detection, which limits the clinical application of this specific marker [10].

In recent years, a new TSI assay, using a pair of recombinant human TSH receptors in a bridging format to capture and detect thyroid-stimulating autoantibodies, has been confirmed by multiple studies to have good diagnostic performance and cost-effectiveness [15, 16]. With the successful development of automated TSI detection kits, the clinical application of TSI in the diagnosis and management of thyroid diseases can be expected [17]. Following a previous registry study of TSI assays conducted in China [18], here we evaluated the efficacy of this automated TSI immunoassay in diagnosing GD and compared it with the third-generation commercial Roche TRAb assay.

## Methods

### Patients

According to a recent study [19] showing that the diagnostic sensitivities of TSI and TRAb were 100% and 94.7% for GD, respectively, the number of GD samples needed to identify the superior effect of TSI in diagnosing GD was at least *n* = 149 (Z-test with α = 0.05, 1-β = 0.9, tests for paired sensitivities in PASS15.0).

We screened 336 consecutive thyroid disease patients and 37 euthyroid controls aged 18–70 years-old from April 2020 to April 2021. The participants were divided into the new-onset GD group (*n* = 227), Hashimoto’s disease (*n* = 29), non-autoimmune thyroid diseases (*n* = 43) (including 29 thyroid nodules, 3 thyroid cancer, 3 idiopathic hypothyroidism and 8 subacute thyroiditis), and euthyroid control (*n* = 37) groups. The diagnosis of GD was based on Chinese thyroid disease diagnosis and treatment guidelines [20] which had the following criteria: the characteristic signs and symptoms of hyperthyroidism, a diffuse goiter, the increased levels of thyroid hormones and decreased levels of TSH. If a patient with thyrotoxicosis was TRAb- and/or TSI-negative, then Doppler ultrasonography and technetium-99 m (Tc-99 m) imaging were performed, and after excluding the possibility of toxic adenoma and nodular goiter, GD was considered. Hyperthyroidism, hypothyroidism, Hashimoto's disease, subacute thyroiditis, thyroid nodules and thyroid cancer were also diagnosed according to the ATA guidelines [8, 20–23]. Participants were excluded if they had a medication history of amiodarone, exogenous thyroxine, glucocorticoids or immunosuppressants, drug use or other abnormal status that could affect the measurement of thyroid function, a recent history of fever, serious systemic disease, or pregnancy. We further excluded participants with missing data on thyroid function and thyroid-related antibodies. This research was approved by the Ethics Committee of Peking university people’s hospital (2021PHB001-001) and was conducted in compliance with the declaration of Helsinki. All patients gave informed consent to participate in this study.

### Laboratory measurements

In our laboratory, the serum concentrations of thyroid hormones and thyroid-related antibodies including thyroid peroxidase antibodies (TPOAb) and thyroglobulin antibody (TGAb) were detected by a supersensitive electrochemiluminescence immunoassay (Siemens healthcare diagnostics K.K.), with reference ranges for: TSH 0.55–4.78 μIU/mL, free thyroxine (FT_4_) 11.45–23.17 pmol/L, and free triiodothyronine (FT_3_) 3.50–6.50 pmol/L, TPOAb < 60 IU/mL and TGAb < 15 IU/mL. TRAb was measured using electrochemiluminescence immunoassay by a Cobas e601 (Roche Diagnostics, Mannheim, Germany). The measuring range for TRAb was 0.30–40 IU/L and the cut-off value provided by the manufacturer was 1.75 IU/L. According to the manufacturer’s quality control, the coefficient of variation (CV) was calculated according to the formula CV = SD/Mean × 100%. The intra-assay CV was 2.29% and the inter-assay CV was 2.65%.

Based on bridge immunoassay technology, automated assay of serum TSI was performed using Siemens IMMULITE 2000 analyzer. It employed a pair of recombinant human TSH receptor constructs in a bridging format which included both the capture and the signal receptor. The captured receptor was formed by replacing the amino acid fragment of the human TSH receptor aa 261–370 with the amino acid fragment of rat luteinizing hormone (LH) or the gonadotropin receptor aa 261–329. The captured receptor was fixed on a microtiter plate and bound to one antibody arm of TSI. The signal receptor, which consists of TSHR (aa 21–261) and secretory alkaline phosphatase (SEAP), binds to the other antibody arm of TSI. The amount of TSI bound was determined by the intensity of enhanced chemiluminescence development by the reaction of SEAP with luminescent substrate. This assay detected TSI with an intra-assay CV of 3.46% and an inter-assay CV of 2.69%, and a CV of 4.34% at a higher TSI concentration (18.9 IU/L) and 4.17% at a lower concentration (0.87 IU/L), respectively. The measuring range for TSI was 0.10–40 IU/L and the cut-off value suggested by the manufacturer was 0.55 IU/L.

### Statistical analysis

Statistical analyses and data processing were performed using the SPSS 24.0 Medcalc19.6 and GraphPad Prism 8 software. Continuous variables were described as the mean ± standard deviation or median (quartile range). Categorical data were described by case number and percentage. The Mann–Whitney U test was used to compare quantitative data between groups. The Chi-square test or Fisher's exact test was used to compare qualitative data between groups. The qualitative consistency of the two detection methods was evaluated by Kappa test and was explained as follows: a kappa value > 0.80 was considered to indicate great agreement, 0.60 to 0.80 substantial, 0.40 to 0.60 moderate, < 0.40 fair. The quantitative consistency between TSI and TRAb detection methods was analyzed using Passing-Bablok regression and Bland–Altman scatter plot after excluding individuals whose test results exceeded the detection limit with TRAb or TSI. The receiver operating characteristic (ROC) curve was drawn using MedCalc and the area under the curve (AUC) and its 95% confidence interval (CI) were calculated. The diagnostic cut-off point was taken as the value of TSI and TRAb at the maximum Youden index (sensitivity + specificity -1). A *P*-value < 0.05 was defined as statistically significant.

## Results

### Characteristics of participants

Totally, data from 336 participants were analyzed in this study, including 227 patients with new-onset GD, 29 with Hashimoto’s disease, 43 with non-autoimmune thyroid disease and 37 controls without a history of thyroid disease. The main characteristics and levels of TSI and TRAb in each group were shown in Table 1. Females were dominant in each group, and the average age of non-autoimmune thyroid disease patients was higher than that of other groups. A TSI > 0.55 IU/L and a TRAb > 1.75 IU/L were determined to be positive according to the manufacturer’s instructions. The positive rates of TSI and TRAb in new-onset GD patients were 96.92% and 95.15%, respectively, which were much higher than those of Hashimoto’s disease, non-autoimmune thyroid disease and control groups.

**Table 1:** Comparison of the main characteristics and levels of TSI and TRAb among groups

	new-onset GD	Hashimoto’s disease	non-autoimmune thyroid diseases	control	P
n	227	29	43	37	
Females/Males (n)	174/53	22/7	31/12	27/10	0.905
Age (y)	39.05 ± 13.27	40.48 ± 10.65	45.16 ± 13.89	37.30 ± 9.96	0.022
TRAb (IU/L)	8.79 (5.09–18.24)	0.31 (0.30–0.46)	0.39 (0.30–0.52)	0.31 (0.30–0.43)	< 0.001
TRAb					
Positive	216 (95.15%)	1 (3.45%)	0 (0.00)	0 (0.00)	< 0.001
Negative	11 (4.85%)	28 (96.55%)	43 (100.00%)	37 (100.00%)	
TSI (IU/L)	7.10 (3.13–16.65)	0.10 (0.10–0.10)	0.10 (0.10–0.10)	0.10 (0.10–0.10)	< 0.001
TSI					
Positive	220 (96.92%)	2 (6.90%)	2 (4.65%)	0 (0.00)	< 0.001
Negative	7 (3.08%)	27 (93.10%)	41 (95.35%)	37 (100.00%)	

Continuous variables were described as the mean ± SD or median (quartile range). Category variables were expressed by number and percentages. Mann–Whitney U test or chi-square test was used to compare the differences among groups

In 227 patients with new-onset GD, the median levels of serum TSI and TRAb were 7.10 IU/L and 8.79 IU/L, respectively. The TSI was positive (> 0.55 IU/L) for 96.92% patients in GD group, except for 7 patients. Similarly, 95.15% of GD patients presented positive TRAb (> 1.75 IU/L), except for 11 patients. In line with expectations, the percentages of non-GD patients that was positive for TSI and TRAb were both very low. The distribution of TSI and TRAb level in each group was shown in Figure S1.

### Correlation between TSI/TRAb and thyroid function

In 210 newly diagnosed GD patients with a simultaneous determination of thyroid function and TSI/TRAb, a positive correlation was shown between the serum levels of FT_4_ and FT_3_ and the titers of TSI and TRAb (for TSI: *r* = 0.243 and *r* = 0.288; for TRAb: *r* = 0.317 and *r* = 0.360; respectively, all *P* < 0.001). The antibody titers arranged by different levels of thyroid function listed as categorical variables in Table 2 also presented a positive association between FT_4_ or FT_3_ and TSI or TRAb. Compared with subjects in the lower quartile levels of thyroid function (FT_4_ < 29.20 pmol/L and/or FT_3_ < 9.60 pmol/L), the percentage of GD patients with upper quartile TSI or TRAb levels was significantly higher in patients with severe hyperthyroidism (FT_4_ > 66.48 pmol/L and/or FT_3_ > 28.51 pmol/L) (all *P* < 0.001).

**Table 2:** TSI or TRAb levels among different degrees of thyroid function status (χ2 test)

	FT_4_ (pmol/L)			FT_3_ (pmol/L)			TSH (μIU/mL)		
	< 29.20	29.20–66.48	> 66.48	< 9.60	9.60–28.51	> 28.51	< 0.003	0.003–0.008	> 0.008
TSI (IU/L)		(N, %)			(N, %)			(N, %)	
< 3.16	21 (40.48%)	28 (53.85)	3 (5.77)	22 (42.31)	28 (53.85)	2 (3.85)	5 (9.62)	27 (51.92)	20 (38.46)
3.16–14.95	14 (13.20)	61 (57.50)	31 (29.25)	13 (12.26)	62 (58.49)	31 (29.25)	19 (17.92)	70 (66.04)	17 (16.04)
> 14.95	17 (32.69)	17 (32.69)	18 (34.62)	17 (32.69)	16 (30.77)	19 (36.54)	11 (21.15)	26 (50.00)	15 (28.85)
	χ2 = 27.061 *P* < 0.001			χ2 = 32.898 *P* < 0.001			χ2 = 11.883 *P* = 0.018		
TRAb (IU/L)									
< 4.86	23 (44.23)	25 (48.08)	4 (7.69)	23 (44.23)	25 (48.08)	4 (7.69)	5 (9.62)	25 (48.08)	22 (42.31)
4.86–17.85	17 (16.04)	63 (59.43)	26 (24.53)	19 (17.92)	63 (59.43)	24 (22.64)	20 (18.87)	69 (65.09)	17 (16.04)
> 17.85	12 (23.08)	18 (34.62)	22 (42.31)	10 (19.23)	18 (34.62)	24 (46.15)	10 (19.23)	29 (55.77)	13 (25.00)
	χ2 = 28.20 *P* < 0.001			χ2 = 30.861 *P* < 0.001			χ2 = 13.606 *P* = 0.009		

Data were expressed as cases (N) and percentage (%)

According to the respective quartiles of TSI, TRAb, FT_4_, FT_3_ and TSH, they were divided into lower quartile groups (< P25), middle quartile groups (P25-P75), and upper quartile groups (> P75), respectively (Table 2). Compared with individuals in the lower quartile TSI and TRAb levels (TSI < 3.16 IU/L, TRAb < 4.86 IU/L), the percentage of FT_4_ and FT_3_ in the upper quartile was significantly higher for those in the upper quartile TSI and TRAb levels (TSI > 14.95 IU/L, TRAb > 17.85 IU/L) (Table 2).

### Comparison of TSI and TRAb assays

**Fig. 1:**
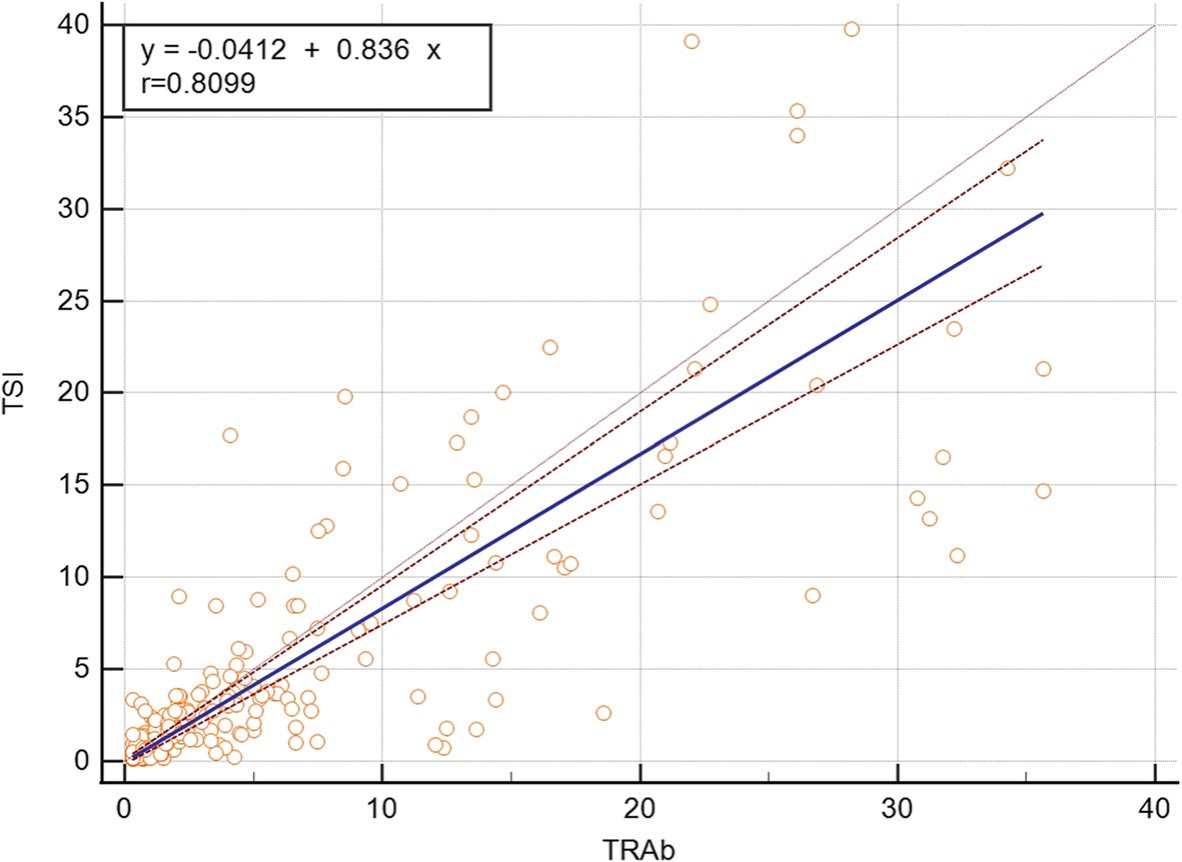
Passing–Bablok regression between TSI and TRAb within the manufacturer-defined measuring range. Correlation coefficient *r* = 0.8099

**Fig. 2:**
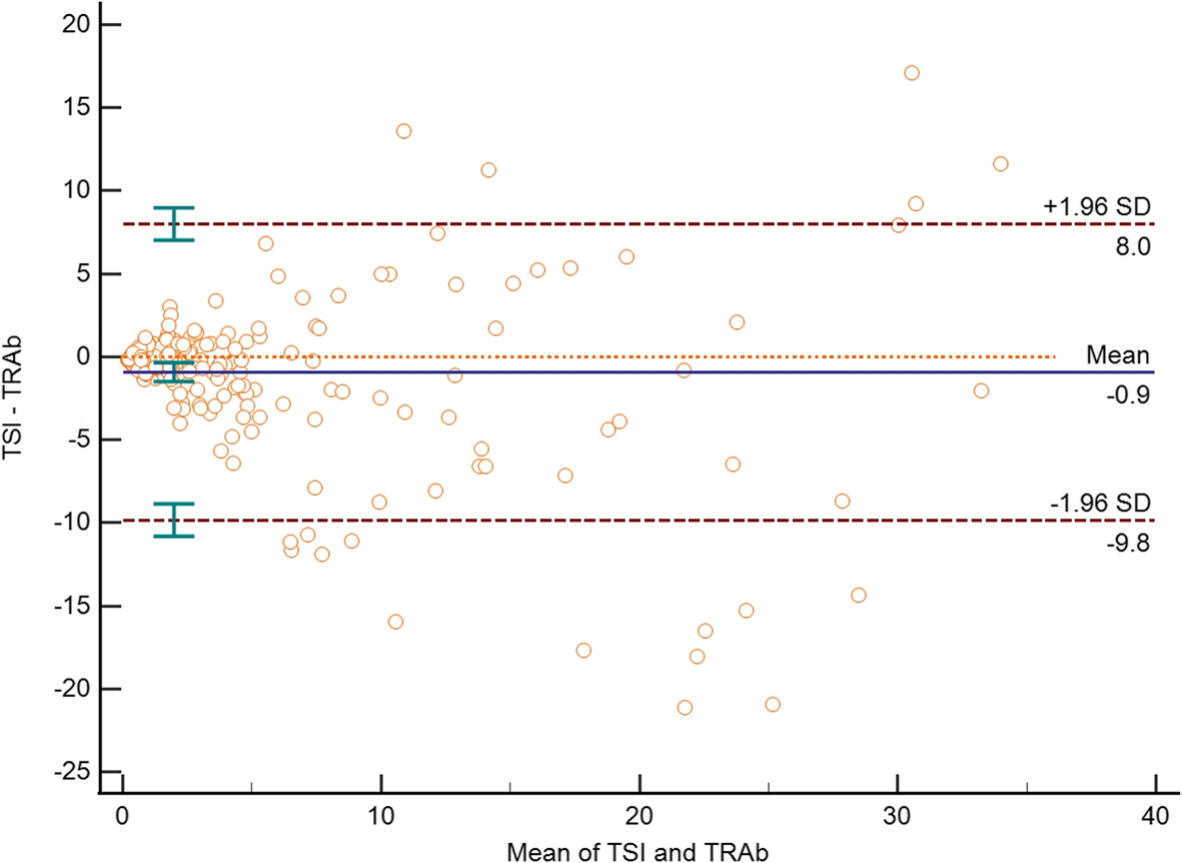
Bland–Altman analysis of TSI and TRAb within the manufacturer-defined measuring range

After excluding 88 individuals whose test results exceeded the detection limit with TRAb (< 0.30 IU/L or > 40 IU/L) or TSI (< 0.10 IU/L or TSI > 40 IU/L), we used Passing-Bablok regression analysis to analyze the consistency of TSI and TRAb detections. The bridge immunoassay TSI positively correlated with TRAb, with a correlation coefficient of *r* = 0.8099 (*P* < 0.0001) (Fig. 1). The Bland–Altman analysis showed a bias of -1.4 IU/L and a total of 15 points (7.15%) exceeded the mean ± 1.96 SD (Fig. 2). Generally, the quantitative agreement between TSI and TRAb was acceptable.

Considering TSI > 0.55 IU/L and TRAb > 1.75 IU/L in the reagent manuals as positive thresholds for GD, the positive and negative coincidence rates for TSI and TRAb was 99.08% and 92.44%, respectively, with a kappa value of 0.93 (Table S1). The sensitivity of TSI and TRAb in GD diagnosis was 96.92% and 95.15%, respectively, showing no significant difference between the two detection methods (*P* = 0.289). Similarly, there was no difference in the specificity of TSI > 0.55 IU/L and TRAb > 1.75 IU/L in diagnosing GD (96.33% vs. 99.08%) (*P* = 0.250) (Table S2). Among 227 newly diagnosed GD patients, 8 presented with discordant qualitative results (6 TSI positive, 2 TRAb positive), indicating that the TSI assay was more consistent with a clinical diagnosis (Table S3). Among patients with new-onset GD, we obtained thyroid ultrasound data from 198 subjects, 68.7% of whom had an enlarged thyroid. Compared with GD patients without goiter, both TRAb and TSI levels were significantly higher in those with goiter (Table S4).

### ROC curve analysis on GD diagnosis

**Fig. 3:**
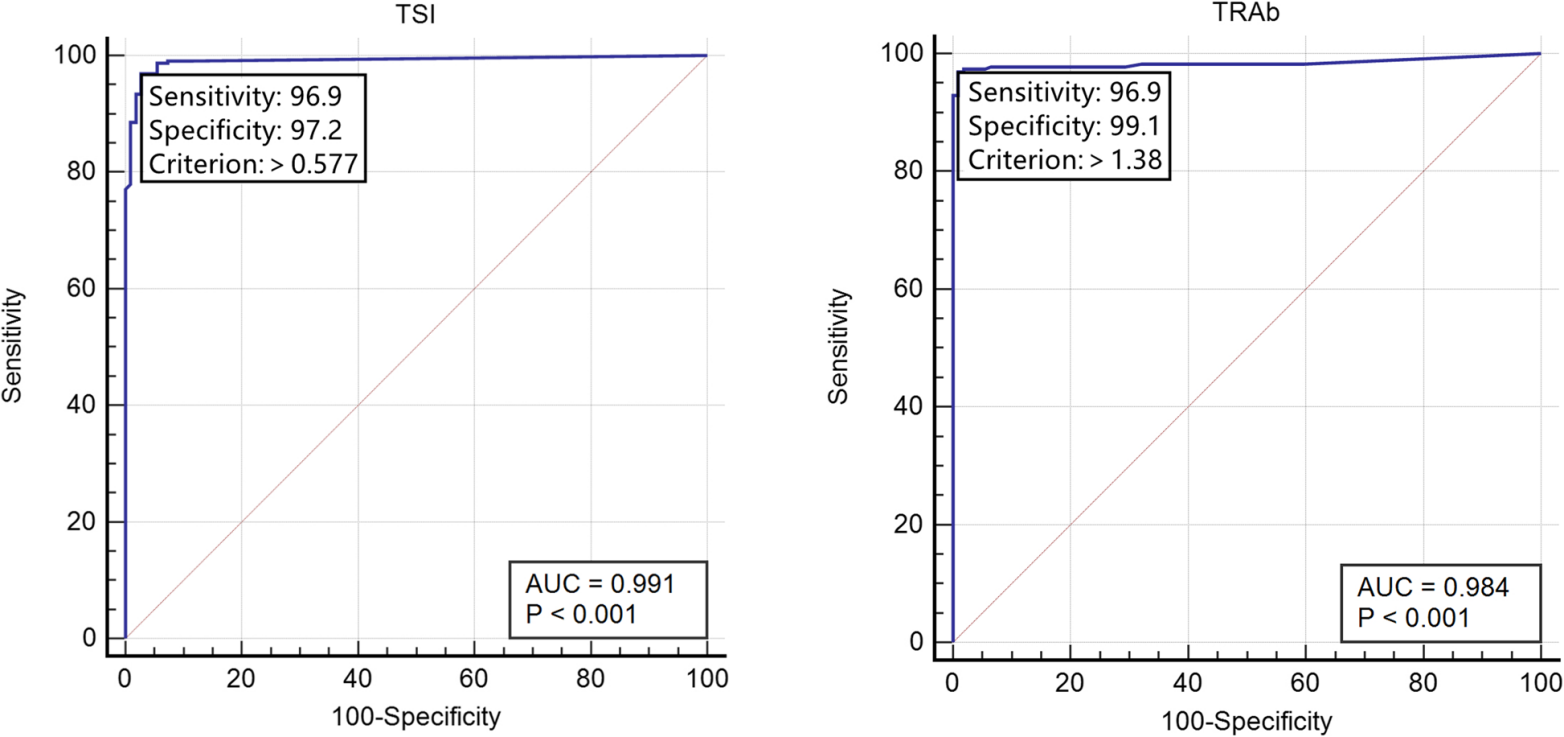
ROC analysis of TSI and TRAb assays to diagnose GD. (**a**) TSI: The area under the curve (AUC) was 0.991. The cut-off value was 0.577 IU/L. (**b**) TRAb: AUC 0.984, cut-off value 1.38 IU/L

The ROC curve (Fig. 3) was drawn with newly developed GD as the dependent variable and TRAb or TSI as the independent variable.

The AUC for the TSI assay was 0.991 (95% CI: 0.974–0.998) and was not significantly different from that for the TRAb assay (0.984, 95% CI: 0.964–0.994) (*P* = 0.2638). We selected the optimal clinical decision-making point (TSI 0.577 IU/L) by the maximum Youden index to distinguish patients with untreated GD from those with other thyroid diseases and control group, with a sensitivity 96.92% (95% CI: 93.70%-98.80%) and a specificity 97.25% (95% CI: 92.20%-99.40%), respectively. A TRAb of 1.38 IU/L was the best cut-off value for GD diagnosis, showing 96.92% sensitivity (95% CI: 93.70%-98.80%) and 99.08% specificity (95% CI: 95.00%-100.00%).

In the current study, there was no significant difference between the new cut-off value for TSI of 0.577 IU/L and the manufacturer-suggested point of 0.55 IU/L in terms of the sensitivity, specificity, and positive rate of TSI in GD patients, Hashimoto's disease patients, non-autoimmune thyroid diseases patients, and healthy controls (*P* = 1.0000). Although there was no significant difference, the sensitivity (96.9%) of the cut-off value for TRAb 1.38 IU/L calculated in this analysis was slightly lower than 1.75 IU/L provided by the manufacturers (95.2%). Among 227 patients with a new clinical diagnosis of GD, 5 were identified as antibody-negative for TRAb 1.75 IU/L. If the TRAb cut-off point of 1.38 IU/L from our analysis was used for re-assessment, 4 of the 5 cases were antibody-positive.

### Comparison of Immulite TSI cut-off points for GD diagnosis

Table 3 showed the TSI cut-off points using a new fully automated chemiluminescent immunoassay (Immulite TSI assay) in previous studies and reagent instructions. The optimal threshold identified to diagnose GD in this study was quite close to the TSI 0.55 IU/L suggested by the manufacturers and 5 other studies, [15–17, 19, 24] excepting the study by Scappaticcio et al. [25]. Compared with the cut-off point of TSI that was obtained in the phase III clinical trial conducted by Cheng et al. [18] in China, there was no significant difference in GD diagnostic efficacy between the two cut-off points (0.42 IU/L vs. 0.577 IU/L).

**Table 3:** Previously published clinical decision points of the TSI assay

Author	Source of subjects	Publication year	Sample size of new-onset GD	Sample size of control	Cut-off points (IU/L)	Sensitivity	Specificity
Frank et al.[17]	Germany	2015	27	325	0.54	99.8%	99.1%
Allelein et al.[24]	Germany	2016	30	221	0.55	100%	98.0%
Tozzoli et al.[15]	Italy	2016	72	311	0.54	100%	98.7%
Villalta et al.[19]	Italy	2018	57	333	0.55	100%	98.2%
Autilio et al.[16]	Italy	2018	46	99	0.57	98.0%	99.0%
Scappaticcio et al.[25]	Switzerland	2020	86	38	0.1	94.2%	84.2%
Cheng et al.[18]	China	2021	100	803	0.42	100%	97.1%
Our study	China	2021	227	109	0.577	96.92%	97.25%

## Discussion

The presence of TSH receptor antibody and the change in its titers have important clinical significance for the diagnostic, treatment and prognostic evaluation of GD. The ATA guidelines suggest that, in patients with hyperthyroidism, especially when there are no typical signs of diffuse thyroid enlargement or orbitopathy, positive TRAb is a simple and effective marker that helps identify GD, and it has a good cost–benefit ratio [8]. Although third-generation automated TRAb detection already has possessed high sensitivity and specificity, it cannot differentiate the types of stimulating or inhibitory antibodies. In contrast, bioassays based on measuring increased cyclic AMP production in cellular systems can specifically detect the levels of TSI, but their use in laboratories is limited due to fussy and time-consuming steps. In recent years, a new automated TSI assay that uses a pair of recombinant human TSH receptors in a bridging format to capture and detect thyroid-stimulating autoantibodies has been developed and has reached the Chinese market China [17]. Here, we evaluated the clinical performance of this new automated TSI immunoassay in comparison with a third generation TRAb assay.

In this study, we recruited participants with hyperthyroidism, Hashimoto's thyroiditis, non-autoimmune thyroid diseases and non-thyroid diseases, covering the spectrum of common outpatients. Quantitative analysis showed that Immulite 2000 TSI and Roche TRAb bioassays had a relatively high correlation (with a slope of 0.999) and excellent concordance (with a 96.73% overall agreement). According to the cut-off value proposed by the manufacturer, the positive rates of TSI and TRAb in new-onset GD patients were 96.92% and 95.15%, respectively, which suggests that the presence of TSH receptor antibody can be used as a key indicator of GD. In line with the results of other studies, the sensitivity and specificity of TSI and TRAb for GD diagnosis were both high and had no significant differences. However, in comparison with TRAb, the qualitative analysis of discordant cases showed that the TSI assay is more consistent with a clinical diagnosis of GD.

The correlation between TRAb or TSI and thyroid functions was controversial in previous studies. Kabadi et al. found that there was no significant correlation between the levels of FT_4_ and TSI in a study using bioassays to detect TSI [26]. In the study by Frank et al.[17], FT_4_ showed a more significant correlation with TSI than with TRAb. In the present study, we found that both TSI and TRAb levels positively correlated with FT_4_ and FT_3_ levels in new-onset GD patients, which is consistent with the results reported by Allelein et al. [24]. The correlation between the TSI/TRAb titers detected by different methods and the degree of hyperthyroidism needs to be explored in a larger number of GD patients in different populations.

Using an ROC curve analysis, a new TSI cut-off value (0.577) IU/L for GD diagnosis with a sensitivity of 96.9% and a specificity of 97.2% was obtained in this study. This new cut-off value was very close to the cut-off value (0.55 IU/L) provided by the manufacturer, as well as the cut-off values reported in several studies (0.54 IU/L, 0.57 IU/L, 0.55 IU/L) [15–17]. Correspondingly, a new TRAb cut-off value of 1.38 IU/L was identified in this study, with a sensitivity of 96.9% and a specificity of 99.1%, which was slightly lower than the cut-off value (1.75 IU/L) proposed by the manufacturer, but was similar to the cut-off value of 1.25 IU/l reported by Tozzoli et al. in an Italian study [27]. If the cut-off value of TRAb obtained in this study (1.38 IU/L) was used for GD diagnosis, the rate of missed diagnosis would be reduced to a certain extent (Table S3). In short, the positive cut-off value of TSH receptor antibody may be different among different studies due to differences in race, sample size, disease status, GD definition criteria, treatment, detection method and the source of the testing kit. Achieving uniform detection and discrimination standards is a key move to incorporate autoimmune antibody indicators into the diagnostic criteria.

After a previous study on the use of the Immulite 2000 TSI assay for GD diagnosis [18], we reported that an automated commercial TSI kit and a positive cut-off value of 0.55 IU/L can be used for routine clinical testing of GD in the Chinese population. Moreover, the concordance rate between TSI detection and clinical GD diagnosis was higher than that of TRAb, which suggests that the fully automated TSI assay will have better application value in the differential diagnosis of hyperthyroidism. This study recruited a large sample of new-onset GD patients, which confirmed the diagnostic efficacy of TSI for GD but also verified the usability of the cut-off points provided by the kit. However, this was a single-center cross-sectional study of new-onset GD patients. Its generality needs to be further verified in more studies using larger populations. The application value of TSI indicators for GD outcome and recurrence prediction also need to be evaluated in future studies.

In conclusion, both TSI and TRAb assays had relatively high diagnostic accuracy for GD. The new-marketed automated TSI detection assay had a higher concordance rate with clinical GD diagnosis and was expected to be promoted in clinical practice.

## Supplementary Information

**Additional file 1:** **Figure S1.** TSI and TRAb levels in the different groups enrolled in the study. GD, Graves’disease; HS Hashimoto’s disease; NATD, non-autoimmune thyroid diseases.

**Additional file 2:** **Table S1.** Qualitative method comparison of TSI and TRAb assays. **Table S2.** Diagnostic consistency analysis of TSI and TRAb for GD

**Additional file 3:** **Table S3.** Description of GD patients with negative TRAb/TSI.

**Additional file 4:** **Table S4.** The association between thyroid volume with TSI/TRAb levels in GD patients.

## Data Availability

The datasets used and/or analyzed during the current study are available from the corresponding author by reasonable request.
