# Supplementary figures and images for: Clinical evaluation of an automated TSI bridge immunoassay in the diagnosis of Graves’ disease and its relationship to the degree of hyperthyroidism
Source: BMC Endocr Disord. 2022 Aug 31;22:218. doi: 10.1186/s12902-022-01114-3 (PMC9429690; doi:10.1186/s12902-022-01114-3)

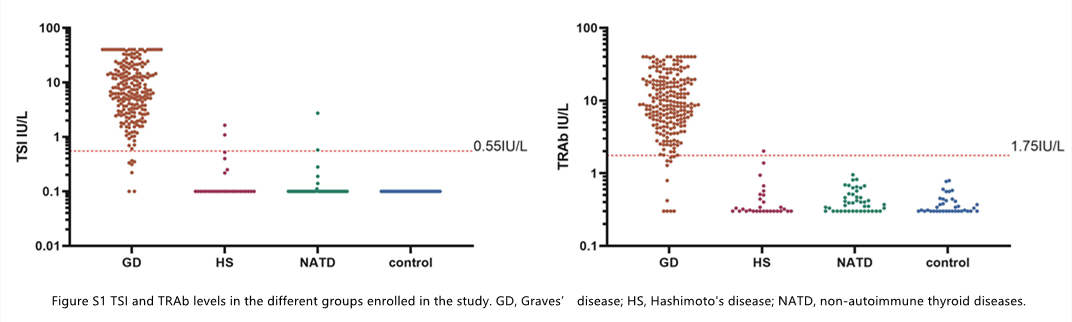

Supplement: Supplementary file 1 — Additional file 1: Figure S1. TSI and TRAb levels in the different groups enrolled in the study. GD, Graves’disease; HS Hashimoto’s disease; NATD, non-autoimmune thyroid diseases. [file 12902_2022_1114_MOESM1_ESM.png]
