# Supplementary material for: Clinical evaluation of an automated TSI bridge immunoassay in the diagnosis of Graves’ disease and its relationship to the degree of hyperthyroidism
Source: BMC Endocr Disord. 2022 Aug 31;22:218. doi: 10.1186/s12902-022-01114-3 (PMC9429690; doi:10.1186/s12902-022-01114-3)
Supplement: Supplementary file 2 — Additional file 2: Table S1. Qualitative method comparison of TSI and TRAb assays. Table S2. Diagnostic consistency analysis of TSI and TRAb for GD [file 12902_2022_1114_MOESM2_ESM.docx]

Table S1 Qualitative method comparison of TSI and TRAb assays.

| TSI | TRAb | | Total |
| --- | --- | --- | --- |
|  | Positive | Negative |  |
| Positive | 215 | 9 | 224 |
| Negative | 2 | 110 | 112 |
| Total | 217 | 119 | 336 |

Data were expressed as cases (N), Kappa test was used to evaluate qualitative consistency between the two detection methods.

Table S2 Diagnostic consistency analysis of TSI and TRAb for GD.

|  | TSI | | |  | TRAb | | |
| --- | --- | --- | --- | --- | --- | --- | --- |
|  | Positive | Negative | Total |  | Positive | Negative | Total |
| GD | 220 | 7 | 227 |  | 216 | 11 | 227 |
| Non-GD | 4 | 105 | 109 |  | 1 | 108 | 109 |
| Total | 224 | 201 | 336 |  | 217 | 119 | 336 |

Data were expressed as cases (N) and percentage (%). The Chi-square test or Fisher's exact test was used to compare qualitative data between groups.
