# Supplementary material for: Clinical evaluation of an automated TSI bridge immunoassay in the diagnosis of Graves’ disease and its relationship to the degree of hyperthyroidism
Source: BMC Endocr Disord. 2022 Aug 31;22:218. doi: 10.1186/s12902-022-01114-3 (PMC9429690; doi:10.1186/s12902-022-01114-3)
Supplement: Supplementary file 3 — Additional file 3: Table S3. Description of GD patients with negative TRAb/TSI. [file 12902_2022_1114_MOESM3_ESM.docx]

Table S3 Description of GD patients with negative TRAb/TSI

| Patient | Gender/Age | FT4  (pmol/L) | FT3  (pmol/L) | TSH  (μIU/mL) | Goiter  degree | TGAb (IU/ml) | TPOAb (IU/ml) | TRAb (IU/L) | TSI (IU/L) | Diagnosis | Decision |
| --- | --- | --- | --- | --- | --- | --- | --- | --- | --- | --- | --- |
| 1 | F/38 | 25.84 | 8.36 | 0.012 | Ⅱ | 269.9 | 219.2 | 0.30 | 0.604↑ | GD | Favour TSI results |
| 2 | M/35 | 18.36 | 6.00 | 0.002 | Ⅰ | N | N | 1.45 | 0.693↑ | GD |  |
| 3 | M/58 | 51.7 | 10.46 | 0.003 | Ⅰ | 192.0 | ＞1300.0 | 1.69 | 0.709↑ | GD |  |
| 4 | M/36 | 44.05 | 11.12 | 0.002 | Ⅱ | ＞500.0 | ＞1300.0 | 1.65 | 1.24↑ | GD |  |
| 5 | F/38 | 48.94 | 14.38 | 0.004 | Ⅰ | 83.4 | 930.8 | 1.28 | 1.53↑ | GD |  |
| 6 | M/30 | 25.87 | 7.56 | 0.002 | Ⅲ | 162.1 | ＞1300.0 | 1.48 | 1.87↑ | GD |  |
| 7 | F/60 | 47.33 | 16.81 | 0.003 | Ⅰ | ＜15.0 | ＜28.0 | 4.25↑ | 0.22 | GD | Favour TRAb results |
| 8 | M/45 | 69.41 | 27.43 | 0.008 | Ⅱ | N | N | 8.18↑ | 0.35 | GD |  |
| 9 | M/66 | 34.79 | 9.28 | 0.003 | Ⅰ | 53.4 | ＞1300.0 | 0.30 | 0.312 | GD | Both TRAb and TSI negative |
| 10 | F/36 | 29.52 | 6.78 | 0.004 | Ⅱ | 286.8 | ＞1300.0 | 0.79 | 0.366 | GD |  |
| 11 | F/32 | 26.25 | 6.77 | 0.007 | Ⅱ | 195.9 | ＞1300.0 | 0.30 | 0.334 | GD |  |
| 12 | F/22 | 28.24 | 8.71 | 0.018 | Ⅱ | 63.6 | ＞1300.0 | 0.42 | 0.10 | GD |  |
| 13 | F/51 | 25.11 | 6.73 | 0.013 | Ⅱ | 23.2 | ＜28.0 | 0.30 | 0.10 | GD |  |
