# Supplementary material for: Clinical evaluation of an automated TSI bridge immunoassay in the diagnosis of Graves’ disease and its relationship to the degree of hyperthyroidism
Source: BMC Endocr Disord. 2022 Aug 31;22:218. doi: 10.1186/s12902-022-01114-3 (PMC9429690; doi:10.1186/s12902-022-01114-3)
Supplement: Supplementary file 4 — Additional file 4: Table S4. The association between thyroid volume with TSI/TRAb levels in GD patients. [file 12902_2022_1114_MOESM4_ESM.docx]

Table S4 The association between thyroid volume with TSI/TRAb levels in GD patients

|  | Without goiter（n=62) | With goiter (n=136) | P |
| --- | --- | --- | --- |
| TRAb | 6.46 (3.28-12.32) | 10.84 (6.35-21.56) | ＜0.001 |
| TSI | 3.52 (1.93-7.61) | 9.61 (5.03-21.05) | ＜0.001 |

Data were expressed as median (quartile range). Mann-Whitney U test was used to compare the differences of TSI/TRAb levels between two groups.
